# The shortlist effect: nestedness contributions as a tool to explain cultural success

**DOI:** 10.1017/ehs.2021.48

**Published:** 2021-11-08

**Authors:** Olivier Morin, Oleg Sobchuk

**Affiliations:** 1 Minds and Traditions Research Group, Max Planck Institute for the Science of Human History. 10, Kahlaische strasse, 07745 Jena, Germany; 2 Institut Jean Nicod, Département d'études cognitives, ENS, EHESS, CNRS, PSL University, UMR 8129. 29, rue d'Ulm, 75014 Paris, France

**Keywords:** Cultural nestedness, nestedness contribution, content-biased selection, cultural evolution, cultural drift, cultural selection, movies

## Abstract

Detecting the forces behind the success or failure of cultural products, such as books or films, remains a challenge. Three such forces are drift, context-biased selection and selection based on content – when things succeed because of their intrinsic appeal. We propose a tool to study content-biased selection in sets of cultural collections – e.g. libraries or movie collections – based on the ‘shortlist effect’: the fact that smaller collections are more selective and more likely to favour highly appealing items over others. We use a model to show that, when the shortlist effect is at work, content-biased cultural selection is associated with greater nestedness in sets of collections. Having established empirically the existence of the shortlist effect, and of content-biased selection, in 28 sets of movie collections, we show that nestedness contributions can be used to estimate to what extent specific movies owe their success to their intrinsic properties. This method can be used in a wide range of datasets to detect the items that owe their success to their intrinsic appeal, as opposed to ‘hidden gems’ or ‘accidental hits’.

## Graphical abstract

**Figure:**
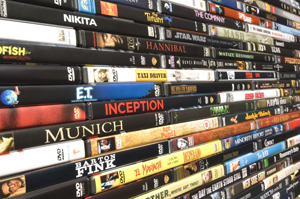

***Social media summary:*** Small movie collections are more selective. We used this to find hidden gems in Netflix and MovieLens data.

## Introduction

The reasons why some cultural products – books, films, songs, artworks – succeed or fail have long been one of the most intriguing questions for the study of culture (Bourdieu, 1993; Heath & Heath, 2007). A common assumption has been that success is meritocratic in nature: canonical authors or award-winning film directors deserve their status owing to the exceptional nature of their work (Bloom, 1994; Galenson, 2007). In recent years, however, an alternative view has been explored, in economics (Frank, 2016), network science (Barabási, 2018) and cultural evolution (Acerbi & Bentley, 2014): a large part of success in culture is due to sheer luck. Recently, it has become possible to approach this understanding using large cultural datasets reflecting human behaviour (Fraiberger et al., 2018; Liu et al., 2018; Yucesoy et al., 2018). In this paper, we apply methods from ecology to detect success based on merit and to separate it from success based on random forces.

Cultural evolution theory offers a useful distinction between three mechanisms potentially leading to success: content-biased cultural selection, context-biased cultural selection and cultural drift (Richerson & Boyd, 2005). Content-biased selection occurs when a cultural item gains in frequency compared with other items because of some intrinsic advantage that makes it more appealing to a wide range of potential adopters (Bell & Sternberg, 2001; Mesoudi et al., 2006; Morin, 2013; Norenzayan et al., 2006; Stubbersfield et al., 2017). Selection based on content is traditionally opposed, on the one hand, to cultural drift, i.e. random and self-reinforcing changes in item frequency, and to selection based on context on the other hand (Henrich & McElreath, 2003). Cultural selection is said to be context-biased when the appeal of items depends not on their intrinsic properties but on local social circumstances, for instance the identity of the persons who adopted these items (prestige-biased selection) or their number (conformity-biased selection).

Drift and context-biased selection are both capable of generating important success differentials between otherwise identical items. Drift results from the fact that agents tend to copy items based on the level of diffusion they have already achieved in a population (what Boyd and Richerson, 1985 call ‘unbiased copying’) with some unpredictable fluctuations. With time, this random process causes some items to get to fixation and others to go extinct, a process that drives all variants but one to extinction, unless new mutations arise regularly. With realistic mutation rates, cultural drift assigns frequencies to cultural items following a power-law distribution, dominated by a few highly prevalent items (Bentley et al., 2004; Leroi et al., 2020; Neiman, 1995). Crucially, these differentials do not reflect any essential difference between items: they arise even if all items are identical in all their intrinsic properties.

Context-biased selection occurs when agents disproportionately copy the cultural choices of the majority (Boyd & Richerson, 1985; Claidière & Whiten, 2012) or those of a prominent individual (Henrich & Gil-White, 2001). Context-biased selection mechanisms generally differ from content-biased ones in that their effects apply irrespective of the content of the items that they target. In this regard context-biased selection is fundamentally similar to drift, and also fundamentally different from content-biased selection, which makes items successful to the extent that they are intrinsically appealing.

While content-biased selection is acknowledged to play a role in cultural evolution, alongside drift and context-biased selection, it is not as thoroughly studied as the other two. Two lines of evidence suggest that content-biased selection cannot simply be assumed. First, neutral models of cultural evolution show that drift may cause cultural items to differ massively in popularity, even if all their attributes are identical (Bentley et al., 2004; Boyd & Richerson, 1985). Second, experimental evidence suggests that drift and conformity, combined, may override content biases. Salganik et al.'s ‘virtual jukebox’ experiment (Salganik et al., 2006) asked participants to rate pop songs by unknown bands, and allowed them to download these songs. In one condition, participants were shown the download figures from other participants, with the most downloaded songs on top. This manipulation increased conformity in download choices and inequalities in song success. It made success less predictable from one group of participants to another, and more unpredictable on the basis of song quality (as assessed by ratings). This study is often interpreted as showing that content matters little to cultural success (e.g. Sunstein and Thaler, 2008), but whether that is the case is debatable. Low-rated songs seldom make it to hit status and songs that did poorly without social influence do not do well when other participants’ choices are highlighted (Acerbi, 2019; Morin, 2016; Salganik et al., 2006). Nonetheless, Salganik et al.'s experiment shows that content biases may be counteracted by a combination of conformity and drift.

Thus, even though there is substantial evidence (experimental and non-experimental) for content-biased selection (Bell & Sternberg, 2001; Berl et al., 2021; Leeuwen et al., 2018; Mesoudi et al., 2006; Norenzayan et al., 2006; Stubbersfield et al., 2017), there is no consensus over the extent to which it impacts cultural evolution. Is it a strong force in its own right, or is it easily overridden – by drift or by conformity-biased selection? This question suffers from a dearth of formal tools that would allow researchers to detect or measure content-biased selection in real-world datasets reflecting ordinary cultural transmission events, where the effects of different evolutionary forces intermingle. This problem is analogous to that of distinguishing between what network science studies as ‘preferential attachment’ (or ‘rich-gets-richer’, an instantiation of the ‘Matthew effect’) and ‘fitness’ (or ‘fit-gets-richer’) (Barabási & Albert, 1999; Pham et al., 2016). When a network evolves, some nodes may attract more links than others because they are already well connected (rich-gets-richer) or because of their intrinsic ‘goodness’ (fit-gets-richer). Network science proposes techniques to tease these two effects apart (Pham et al., 2016), but they require good time series data, which is not always available.

This paper takes a different approach. We claim that content-biased selection has an effect on the distribution of cultural items in collections of varying sizes. More precisely, smaller collections are more selective than bigger collections: their owners are more likely to prefer highly appealing items, having less room to include less appealing ones. This effect – the ‘shortlist effect’, as we call it – is one form of content-biased selection, but content-biased selection can occur independently of it. The shortlist effect results from the fact that some collections are smaller than others, forcing their owners to be more picky. We only expect the shortlist effect to obtain under certain conditions. First, some form of cultural selection has to be at work. Furthermore, collection size has to be exogenously determined to some extent. In other words, the size of a collection is not simply a function of how many times an agent has been exposed to items that she liked. There must have been times when the agent encountered items that she would have chosen had it not been for some external limitation.

The shortlist effect, when it occurs, has two consequences. First, the shortlist effect causes content-biased selection to increase the nestedness of sets of collections of cultural items. Second, we can estimate the degree to which an item is subject to cultural selection from knowing the contribution that it makes to the nestedness of a dataset. This paper does three things to ground these claims. We use a model to show that, when the shortlist effect is at work, cultural selection is associated with greater nestedness in sets of collections. We establish empirically the existence of the shortlist effect in 28 sets of movie collections. We then use this same data to show that nestedness contributions can be used to estimate to what extent specific movies owe their success to their own appeal.

**Figure 1.**
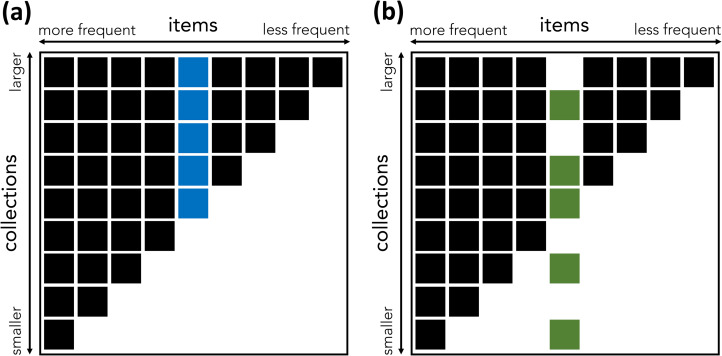
An illustration of the concepts of nestedness and nestedness contribution. (a, b) Two binary matrices representing items in collections. A box indicates that the relevant item (column) is present in the relevant collection (row). Matrix (a) has higher nestedness than matrix (b), because of the distribution of the colored item. In (a), the blue item is present in all large collections above a certain threshold, and the matrix is perfectly nested: the items found in any small collection are a proper subset of the set of items found in any bigger collection. In (b), the green item, which has the same frequency as the blue item, is present in some, but not all, of the biggest collections, and also in some of the smallest. As a result, (b) is not perfectly nested: some of the bigger collections miss some of the items present in smaller ones. The blue item contributes more to the nestedness of (a) than the green item contributes to the nestedness of (b). (Inspired by Saavedra et al., 2011.)

Nestedness is a characteristic of some bipartite networks. In bipartite networks, nodes of one type project to nodes of another type. Bipartite networks are often used in ecology to model the presence of species (node type A) in ecosystems (node type B) or the interaction of pollinating species with flowering plants (Mariani et al., 2019; Patterson, 1987). Typical examples of nestedness are found in ecosystems – the species found in low-diversity biotopes tend to also be found in high-diversity biotopes; in pollination networks – specialist plants are pollinated by generalist insects; in global economies – the countries that export few products tend to export goods that are also exported by countries with more diversified exports (Bustos et al., 2012; Mariani et al., 2019). A cultural equivalent of nestedness can be found in collections of cultural items (Figure 1). What we call a collection is a repertoire of items associated with an individual or a group (e.g. a collection of technologies in a particular society, or the books in a personal library). ‘Items’ can be any kind of cultural product (e.g. technologies, books). Looking at the technologies present in various human cultures, Kamilar and Atkinson (2014) find a substantial degree of nestedness: the technologies possessed by less technologically complex cultures tend to be also possessed by more complex ones. This finding was foreshadowed by the humanities scholars who found that the books found in small libraries tend to be a subset of the books found in bigger libraries (Chartier, 1982; Moretti, 1998).

A pervasive phenomenon, nestedness defies general explanations. In the case of cultural nestedness in technological repertoires, Kamilar and Atkinson (2014) argue that nestedness is a mark of cumulative cultural change, i.e. the elaboration of novel technologies based on existing ones. In technologically rich cultures, the technologies of less complex cultures co-exist with the novel technologies they were elaborated from, resulting in a nested pattern. This stimulating hypothesis cannot, however, explain cultural nestedness in other domains that do not show cumulative progress, like book collections.

Cumulative changes like those posited by Kamilar and Atkinson are not, we contend, the only possible cause for cultural nestedness. Nestedness may also occur as a result of content-biased selection, among other mechanisms. We use a simple model of cultural collections to show, first, that some amount of nestedness naturally occurs when some collections are more capacious than others, and some items are more frequently encountered; second, that content-biased cultural selection can increase nestedness, provided that small collections are more selective than bigger ones. Then we use two datasets of film collections, divided into 28 subsets, to establish the shortlist effect, and to show that the movies most subject to cultural selection tend to make bigger contributions to the dataset's nestedness.

## Selection and nestedness in cultural collections

The shortlist effect is the tendency for selection based on content to be stronger when an agent has fewer resources to devote to the acquisition of cultural items: a short list of candidates is more selective than a long list. Acquiring cultural items takes resources, the nature of which can vary: time and effort to learn a technique or a tale, space to store artworks or books, money to see a movie, etc. Small collections should prefer appealing items, while bigger collections should contain both appealing and less appealing items. Under this assumption, content-biased selection should increase the nestedness of cultural collections. Content-biased selection brings the frequency of cultural items (the number of collections where an item figures) in line with their intrinsic appeal (the extent to which their content makes people want to add them to their collection). If content-biased selection is high, appealing items become frequent. As a result of the shortlist effect, these appealing and frequent items are more likely to be found in small collections. This increases nestedness, because it increases the probability that the items present in small collections are also included in bigger collections.

The constitution of cultural collections can be modelled with equation (1):1
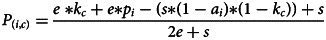
where *P_(i,c)_* is the probability that item *i* is present in collection *c*, *p_i_* and *a_i_* are the prevalence and appeal of item *i*, *k_c_* is the capacity of collection *c*, and *e* and *s* are constants. The first term of the equation, e*k_*c*_, ensures that collections with a higher capacity are more likely to acquire any item. It models the fact that some collections are bigger than others simply because their owners devote more resources to collecting items. The second term, e*p_*i*_, ensures that more prevalent items are more likely to become more frequent. It is meant to model the fact that some items are more widespread in a population, and thus more likely to be encountered and adopted (a key premise of both drift and conformity-biased selection). The last term of the equation models the shortlist effect. It increases the probability that high-appeal items will be present in a collection, but only to the extent that the collection has low capacity. The constant s modulates this effect. We used equation (1) to simulate several series of cultural collections. Under a broad range of assumptions, we found that sets of cultural collections were significantly nested.

To measure nestedness, we used NODF (Nestedness metric based on Overlap and Decreasing Fill), a tool first proposed by Almeida-Neto et al. (2008), which then became the most commonly used metric for assessing nestedness. By sequentially measuring the overlap between the pairs of rows and columns in a matrix, NODF allows us to put a precise number (between 1 and 100) on the triangular pattern typical of a nested system. (See Methods.)

Our model reveals that nestedness can emerge from the simplest conditions and does not require cumulative cultural progress in the sense implied by Kamilar and Atkinson (2014). Even when *e* is 10^6^ fold higher than *s*, so that the shortlist effect is entirely negligible, we find important and significant levels of nestedness (all NODFs > 60, all values of *p* < 0.005).

**Figure 2.**
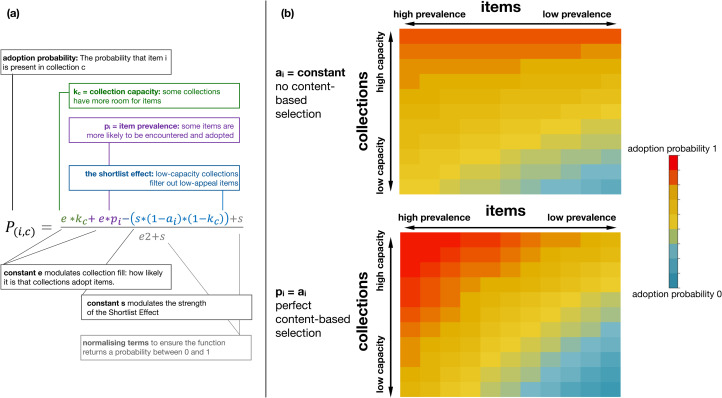
A model of cultural collections predicts that content-biased selection increases the nestedness of a set of collections. (a) Our model is built upon the adoption probability function, which models the probability that an item will be added to a collection, based on the collection's capacity to adopt items, the item's prevalence, and its appeal (all represented by values between 0 and 1). (b) We ran this equation for 11 items and 11 collections of varying capacity and prevalence, changing only the value of the correlation between appeal and prevalence. In the first simulation (top panel), appeal is constant and independent of prevalence (no content-biased selection). In the second simulation (bottom panel), appeal is identical to prevalence (perfect content-biased selection). The resulting pattern of adoption probabilities is consistent with high nestedness: the items in low-capacity collections are likely to be a subset of the items present in high-capacity collections. Low-prevalence items do not occur in low-capacity collections, and high-prevalence items are very likely to occur in high-capacity collections. Simulations were computed with *e* = *s* = 1. Changing the values of *e* or *s* does not change the qualitative pattern shown here (see Methods).

How does content-biased selection modulate nestedness? Content-biased selection can be defined, for our purposes, as the correlation between item prevalence and item appeal. It is maximal if item prevalence closely tracks item appeal, minimal if there is no correlation between the two, and inverse if an item's prevalence is negatively predicted by its appeal. Content-biased selection should increase nestedness by increasing the presence of high-frequency items and decreasing the presence of low-frequency items in small collections (Figure 2).

Simulations confirm this intuition. For a broad range of values of *e* and *s*, nestedness (measured with NODF) increases as the correlation between prevalence and appeal gets higher. This relation breaks down when the shortlist effect is cancelled because *s* is negligible compared with *e* – 50 times lower or more (see Methods and Table 1). Intuitively, when *e* is much bigger than *s,* collections are highly likely to acquire new items in a way that is not modulated by the shortlist effect. In such cases, where the shortlist effect has a negligible impact, we do not expect selection to impact nestedness – and it does not. Content-biased selection drives nestedness through the shortlist effect. The shortlist effect means that low-appeal items are relegated to big collections only, and selection brings frequency in line with appeal. Low-appeal items are also low frequency, and vice-versa. When low-frequency items are present in big collections only, that is nestedness. When high-frequency items are present in all collections big or small, that is also a manifestation of nestedness.

**Table 1.** Correlation between content-biased selection and nestedness: results of the simulations. Each cell shows the result of 5 times 30 simulations of a 200 × 200 binary matrix. The matrix was populated using the adoption probability function (equation 1). The values that the parameters *s* and *e* were given in the equation change from cell to cell. Each of the 200 items was given a frequency *k* and an appeal *a*. The correlation between frequencies and appeals, representing content-biased selection, was 0, 0.25, 0.5, 0.75 or 1. We considered how the nestedness of the matrix was correlated with the value of content-biased selection. Each cell shows the correlation between nestedness (NODF values) and content-biased selection. The correlation was measured over five data points, each data point representing the average NODF measured over 30 simulations for one level of content-biased selection (out of five). Inside each cell, the left-side figure is the raw correlation, the right-side figure after the slash is the partial correlation controlling for matrix fill, both rounded to the lower value. All cells where the partial correlation is >0.9 are in bold. A tight correlation between nestedness and content-biased selection holds whenever *e* is not much higher than *s*

	Values of *s*								
Values of *e*		**0.0000001**	**0.001**	**0.01**	**0.1**	**0.25**	**0.5**	**0.75**	**1**
	**0**.**0000001**	**0.99**/**0.99**	**0.97**/**0.90**	0.94/0.87	**0.88**/**0.90**	0.88/0.73	**0.93**/**0.94**	**0.92**/**0.90**	0.85/0.84
	**0**.**001**	0.91/0.69	**0.99**/**0.99**	**0.99/0.99**	**0.99**/**0.99**	**0.97**/**0.97**	0.82/0.85	0.75/0.86	0.91/0.71
	**0**.**01**	0.75/0.70	0.92/0.77	**0.99/0.99**	**0.99**/**0.99**	**0.99/0. 98**	**0.99**/**0.98**	**0.98**/**0.94**	0.96/0.83
	**0**.**1**	0.76/0.79	0.72/0.26	**0.99/0. 94**	**0.99**/**0.99**	**0.99**/**0.99**	**0.99**/**0.99**	**0.99**/**0.99**	**0.99**/**0.99**
	**0**.**25**	-0.15/0.26	0.40/0.34	0.01/-0.42	**0.99**/**0.99**	**0.99**/**0.99**	**0.99**/**0.99**	**0.99**/**0.99**	**0.99**/**0.99**
	**0**.**5**	0.87/0.68	0.93/0.92	-0.71/0.32	**0.99**/**0.99**	**0.99**/**0.99**	**0.99**/**0.99**	**0.99**/**0.99**	**0.99**/**0.99**
	**0**.**75**	0.07/-0.32	0.54/0.55	0.90/0.24	0.98/0.89	**0.99**/**0.99**	**0.98**/**0.99**	**0.99**/**0.99**	**0.99**/**0.99**
	**1**	0.59/0.58	-0.04/0.38	-0.42/0.54	**0.98**/**0.96**	**0.99**/**0.98**	**0.99**/**0.97**	**0.98**/**0.99**	**0.99**/**0.99**

## The shortlist effect and its consequences in movie collections

The nestedness of a set of collections is determined by many things, like the sizes of collections and their distribution, or item frequencies and their distribution. These confounds mean that, by itself, the nestedness of a naturally occurring set of collections reflects a host of processes distinct from the one we are interested in. What the model above suggests, however, is that nestedness could be used to study content-biased selection on specific items. Just like items under selection contribute to nestedness (see above), we expect the items which escape selection to make a lesser contribution to nestedness. Items that elude selection are of two types: ‘hidden gems’ that have high appeal but low frequency, and ‘accidental hits’ that have low appeal but high frequency. If the shortlist effect is at work, both kinds of items should reduce the nestedness of a set of collections. Hidden gems are appealing: the shortlist effect will push them into small collections, even though they are infrequent. This is a departure from nestedness, since, under perfect nestedness, low-frequency items are only present in the biggest collections. Likewise, accidental hits disrupt nestedness. They are low in appeal, but high in frequency. The shortlist effect will keep them out of some small collections where perfect nestedness would require them to be present.

We explore this hypothesis using large datasets of movie collections (the Netflix prize dataset: Bennett et al., 2007; the MovieLens dataset: Harper & Konstan, 2015). *Collections* are sets of movies rated by users of a web platform (MovieLens or Netflix). All of the movies that a given user has rated constitute her ‘collection’. A movie's *frequency* is the number of users who rated this movie. It is identical to the number of collections where the movie figures (no user has more than one collection).

We considered the contribution that individual movies make to the nestedness of a set of collections. A movie's nestedness contribution (after Saavedra et al., 2011) is obtained by considering the average nestedness of a set of collections where real data for the item's presence or absence in the collections has been randomised (see Methods).

We divided our two datasets into 28 sets of movie collections overall, defined by genre (e.g. ‘horror’, ‘drama’, ‘romantic comedy’). The first dataset consisted of 15 genre-defined sets of collections from the MovieLens database. The results we found on this dataset were arrived at in an exploratory fashion, while testing a slightly different hypothesis. Thus, we decided to run a preregistered replication on a different dataset, the Netflix dataset, which was also broken down into 13 genre-defined datasets.

Both the MovieLens and the Netflix datasets are lists of ratings. We treated all of the movies rated by a user as that user's collection. This is of course an approximation, since users could rate movies they have not seen, and (more likely) see movies without rating them. To limit the consequences of this potential bias, we removed the users who rated only a small number of movies (see Methods). We used the values of users’ ratings as a proxy to measure appeal. Web ratings on websites like Internet Movie Database (IMDb: www.imdb.com), or Metacritic (www.metacritic.com) correlate well with the popularity and significance of movies (Wasserman et al., 2015). Four sources of ratings were used overall. Two sets of ratings were directly sourced from the Netflix and MovieLens datasets. The other two, sourced from the Metacritic or IMDb websites, were completely independent: they were provided by different users, under different conditions. The MovieLens dataset was analysed with three sets of ratings: MovieLens, Metacritic and IMDb. For the replication on the Netflix dataset, we dropped Metacritic ratings (which do not cover enough movies), replacing them with Netflix ratings. We verified that the data met our theoretical assumptions for all genre subsets and all relevant rating sources. All genre subsets showed substantial and significant nestedness (NODF values between 40 and 79 for MovieLens, between 31 and 45 for Netflix, all significant at the *p* < 0.005 level – see Methods on significance testing for NODF). NODFs are lower for the Netflix datasets because more low-frequency movies were included in those (see Methods), and NODF is sensitive to matrix fill (Almeida-Neto et al., 2008).

For the purpose of this study, we define content-biased selection as a positive correlation between movie ratings and movie frequencies. There was content-biased selection, in this sense, in all 28 genre subsets, in line with other studies of web ratings (Acerbi, 2019; Wasserman et al., 2015). The raw correlation between movie frequency and movie ratings (Spearman's rho) is positive no matter what source is used for the ratings (Supplementary Tables S3 and S4).

**Figure 3.**
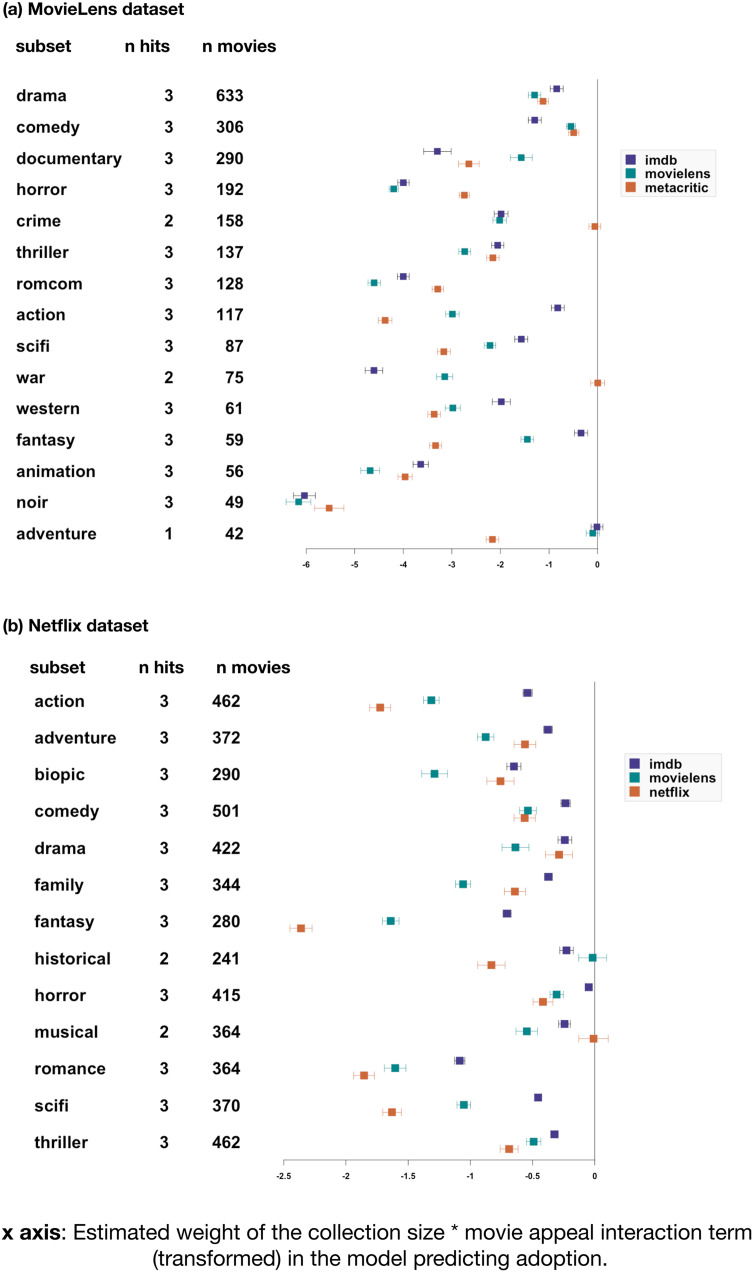
The shortlist effect: highly rated movies are more likely to enter smaller collections. Results of the test of our prediction on the MovieLens (a) and Netflix (b) datasets. Genre subsets are listed on the *y*-axis (first column). Each subset was analysed three times, for each of three different sources: MovieLens and IMDb for both datasets, with the addition of Metacritic ratings (for MovieLens data) and Netflix ratings (for Netflix data). The second column (‘*n* hits’) shows how many of the three tests verified the prediction. We considered the test to be a hit when the addition of an interaction term for appeal modulated by collection size made the model more informative (Δ_AIC_ > 2), and when the sign of the interaction term was the opposite to the estimated weight of appeal. The third column indicates how many individual movies the regression was computed on. The graph represents each model's estimate for the interaction term, modified as follows: if the main effect of appeal over adoption in a collection is negative, we report the negative of the interaction term (if a value is positive, it is transformed to negative). If the main effect of appeal over adoption in a collection is positive, the interaction term is plotted as it is. Effect sizes are typically much larger for the MovieLens dataset (top panel) compared with the Netflix dataset, probably owing to the broader range of collections and movies (including large numbers of very small collections and highly unsuccessful movies) in the Netflix dataset. Error bars indicate 95% confidence intervals.

We found a robust shortlist effect. Highly rated movies were more likely to be present in small collections, controlling for their frequency. For all genre subsets we built a logistic binomial regression to predict whether a given movie was present in a given collection, using the movie's frequency (i.e. how many collections in the subset contain it), its appeal (as given by one of the rating sources) and the collection's size. This formed the ‘baseline model’. All variables were normalised using min–max normalisation to bring their value between 0 and 1, and avoid variable-scaling issues. Such normalisation is questionable for ratings, so we replicated all the analyses described below without normalising the ratings. This did not change the pattern of results described below in any substantial way (see the online open data and code). We added to our baseline model an interaction term for collection size × appeal, resulting in the test model. We considered that the shortlist effect obtained when the test model proved more informative than the null model (Δ_AIC_ > 2), and its estimate for the interaction term ran in the opposite direction to the effect of appeal. If the main effect of appeal is positive, then a negative interaction term validates our prediction: the positive effect of appeal is lower in large collections. Conversely, if the main effect of appeal is negative, then a positive interaction term validates our prediction: the negative effect of appeal is lower in small collections. In other words, smaller collections are more selective for high-appeal items. When the effect of appeal is negative, the interaction term is positive, meaning that bigger collections are less biased towards high-appeal items; when the effect of appeal is positive, the interaction term is negative, which amounts to the same thing – smaller collections are more biased towards high-appeal items, thus bigger collections are less biased towards high-appeal items. This prediction was verified in 13–14 genre subsets out of 15 (depending on the ratings’ source) for the MovieLens datasets, and 12–13 out of 13 for the Netflix dataset (Figure 3). Re-running this analysis with mixed-effects models that include a random effect for movie and one for collection replicates these results, with 14–15 genre subsets verifying the prediction (depending on the ratings’ source) for MovieLens, and 11–13 out of 13 for the Netflix set.

**Figure 4.**
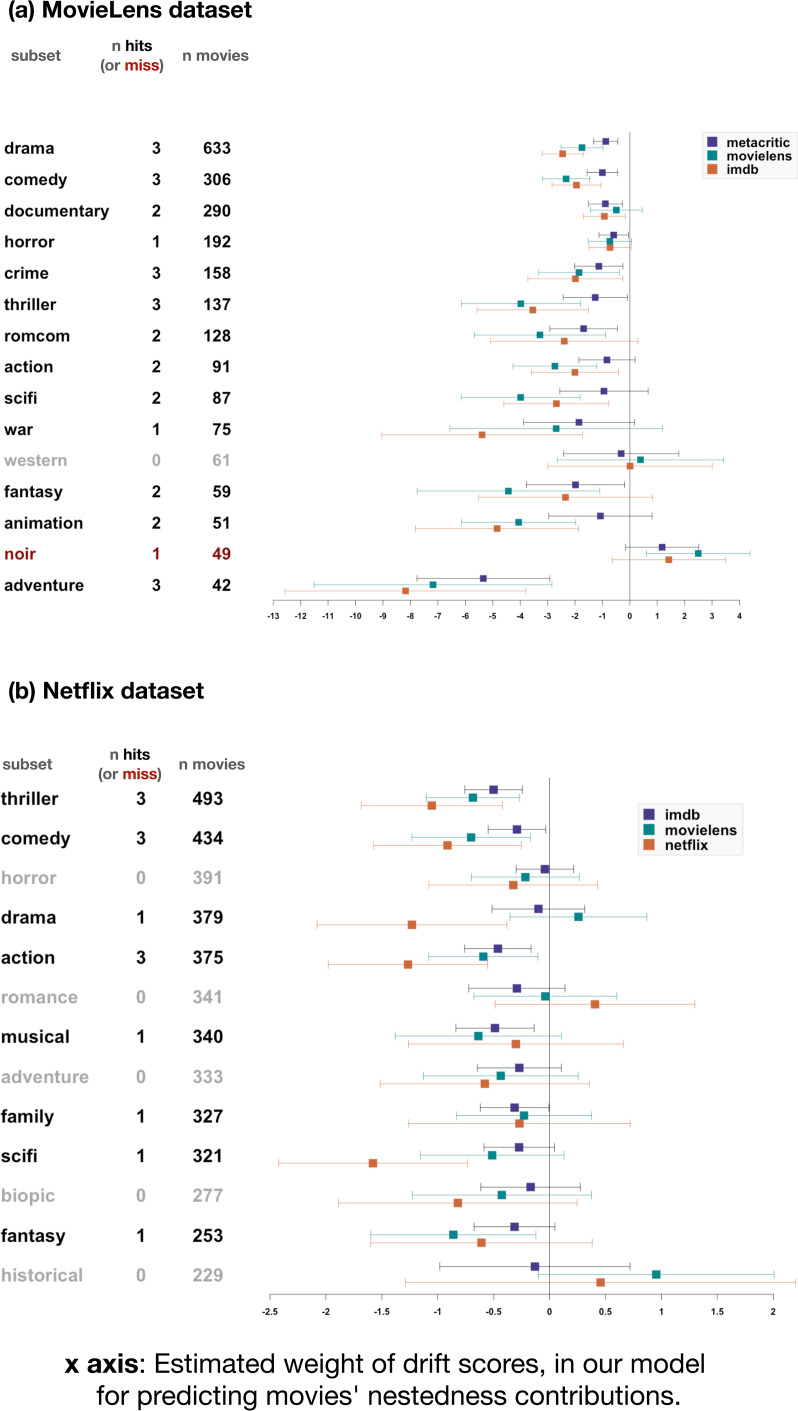
Drift scores negatively predict nestedness contributions. Estimates for the weight given to movies’ drift score in our nested regression model predicting movies’ nestedness contributions, for the MovieLens (a) and Netflix (b) dataset. Genre subsets are listed on the *y*-axis (first column). Each subset was analysed three times, for each of three different sources of ratings: MovieLens and IMDb for both datasets, with the addition of Metacritic ratings (for MovieLens data) and Netflix ratings (for Netflix data). The second column (‘*n* hits’) counts how many of the three tests confirmed our hypothesis (‘hits’) or reject it (‘miss’). We considered the test to be a hit when the model including drift scores was more informative than the baseline model (Δ_AIC_ > 2) and estimated the weight of drift scores to be negative. Error bars indicate 95% confidence intervals.

We then tested our main prediction: items subject to strong content-biased selection should make a greater contribution to nestedness. In each subset, we first built a linear regression model to predict each movie's rating using its frequency. We then took the residuals from this regression, and transformed all negative residuals to positive values. The resulting measure captures the extent to which actual ratings are distant from those that would be predicted, assuming a perfect correlation with frequency – what we informally call a ‘drift score’. This drift score was then used in a series of linear regressions: first, a regression model predicting nestedness contribution on the basis of movie frequency; then, adding year of release as a second predictor if it proved more informative (in most cases, it did); lastly, adding our ‘drift score’ and measuring whether this made the model more informative (using a threshold of Δ_AIC_ > 2). Although drift score and frequency tend to be correlated, multicollinearity remains tolerably low (variance inflation factor < 1.6 for all datasets, subsets and rating sources). For the MovieLens dataset, our prediction was confirmed for at least one source of ratings out of three in 13 genre subsets out of 15. Only in one subset (‘film noir’ movies) do we find the opposite pattern. For the Netflix dataset, the results are more subtle, probably due to the fact that our preregistered inclusion criteria let in a large number of movies rated by only a few users. Nevertheless, in most genre subsets (eight out of 13), our prediction is validated for at least one source, and it is never refuted (Figure 4).

A movie gets a high drift score if it is under-rated or over-rated, given its frequency. If it is under-rated, it is present in many collections in spite of its low ratings. If over-rated, the opposite is true. Our findings show that such under- or over-rated movies tend to make a lesser contribution to the nestedness of sets of movie collections. Conversely, movies contribute more to the nestedness of a set of movie collections when their frequency in the population is aligned with their appeal (as indexed by the ratings they get).

## Discussion

If and when content-biased selection is stronger in smaller cultural collections (the shortlist effect), content-biased selection should increase the nestedness of a set of collections. We validated a prediction derived from this hypothesis, by showing that the movies whose frequency matches their appeal tend to make a bigger contribution to the nestedness of sets of collections. This study provides additional evidence for the little-studied phenomenon of cultural nestedness (Kamilar & Atkinson, 2014) and highlights content-biased selection as one possible source of it. Our model showing that content-biased selection, combined with the shortlist effect, can increase nestedness in cultural collection highlights the multi-causal nature of nestedness. It suggests that high nestedness on its own (as measured, for instance, by high NODF scores) cannot be used to infer specific processes in cultural data (like cumulative culture or content-biased selection), since many different factors influence nestedness. We then proceeded to test two empirical claims on empirical datasets: the shortlist effect – small collections are more selective for high-appeal items – and the negative correlation between a movie's ‘drift score’ (measuring the discrepancy between a movie's appeal and its success) and its contribution to the nestedness of its corpus. We found the results to be consistent with both predictions.

The main limitation of our study has to do with the measurement of appeal and of appeal-biased selection. Neither can be measured directly with our data, so we use proxy measures to assess them. Cultural evolutionists, when studying content-biased selection, often use a shortcut definition, under which selection takes place whenever a cultural trait is more likely to be acquired than another trait (Mesoudi, 2021). We do the same here. This shortcut definition allows us to measure selection as the correlation between frequency and appeal, in line with other studies of content-biased selection (see, e.g. Norenzayan et al., 2006). However, a more complete definition of selection would require us to measure the ‘fitness’ of movies over time, across multiple episodes of adoption and retransmission. Such an approach would be more faithful to biological models of selection, but it would not necessarily reflect the way movies gain viewers. Word-of-mouth plays some role in movie diffusion, but other channels (advertisement, traditional media, etc.) play a major role too. Standard selectionist dynamics, where the prevalence of a trait depends on its decentralised transmission from one agent to another, may thus not apply here. Cultural selection, here, as in other cases (Claidière et al., 2014), is far from a perfect analogue of natural selection.

Another limitation concerns the validity of ratings as indicators of intrinsic appeal. A movie is intrinsically appealing when the average viewer is likely to appreciate it because the movie is enjoyable in its own right, not because of the other viewers who viewed or rated it. The word ‘intrinsic’ does not have any deeper meaning here. Appeal is in the eye of the viewer, and often hinges on superficial features (the popularity of the lead actor, the language being used, etc.). A movie is intrinsically appealing if the average viewer is likely to enjoy it independently of how many other viewers rated it.

Ratings are only a proxy for appeal: raters may have idiosyncratic tastes, or simply hit the wrong button, reviews can be fabricated, etc. Appeal is such a subjective thing that it seems measurable only indirectly – so the external validity of ratings is difficult to establish, except by comparing them with other ratings. Fortunately, several cues lead us to think that the ratings used here escape at least some of the criticisms that online ratings are exposed to (de Langhe et al., 2016; Hu et al., 2006; Koh et al., 2010). Online ratings generally suffer from two main biases (Hu et al., 2006). The acquisition bias means that people rate a product once they have expressed a preference for it (by purchasing it or, here, by viewing it). Raters are thus favourably disposed. The under-reporting bias prevents people with a moderate opinion from giving a rating: people are much more likely to rate an item if they have a strong opinion about it – be it positive or negative. While the acquisition bias produces an excess of positive ratings (i.e. a negative skew), the under-reporting bias can result in U- or J-shaped distributions where an item gets some very bad ratings, many good ratings, but few ratings in the middle. How susceptible are movie ratings to these effects? Koh et al. (2010) show that the answer varies depending on websites and rating systems. In their analysis, IMDb rating distributions present clear signs of an under-reporting bias against moderate opinions, but that is not at all the case for MovieLens ratings. Hu et al. (2006) explain this by noting that MovieLens users were incentivised to rate many movies in order to unlock movie recommendations (Harper & Konstan, 2015). We replicate their observation: our MovieLens ratings are normally distributed with no evidence of an under-reporting bias. The same finding obtains for Netflix ratings (Supplementary Materials, Figure S1).

Another reason to trust ratings as informative indicators is their relative consistency across sources. Depending on the dataset and corpus, we find that the agreement between our three sources of ratings (IMDb, MovieLens, and Metacritic for the MovieLens dataset; IMDb, MovieLens, and Netflix for the Netflix dataset) is moderate to good. The intra-class correlation values, reflecting the share of the variance captured by movies as opposed to rating sources, ranges (depending on the corpus) between 0.59 and 0.78 (for the Netflix dataset) or between 0.55 and 0.76 (for the MovieLens dataset) (Supplementary Tables S5 and S6; note that none of the analyses presented here assumes any kind of consistency between rating sources, since all predictions were tested separately on each source of ratings). More work is certainly needed to explore the relationship between ratings and intrinsic appeal, but the fact that ratings behave according to our three predictions (the correlation between ratings and frequency, the shortlist effect and the correlation between drift scores and nestedness contributions) encourages us to treat them as proxies for intrinsic appeal.

The shortlist effect, showing that the advantage of appealing items is modulated by collection size, provides us with a novel way of studying cultural selection. The range of possible factors that can put an exogenous limit on collection size and trigger the shortlist effect is quite broad. They can be material (a lack of space to store books, a lack of money to buy them), institutional (the shortlist for a literary award is restricted by convention) or cognitive (the limitations of memory and attention). In the case of the lists of movies studied here, the only hard constraint that limited the size of a user's collection was the amount of time they were willing to spend rating movies online. Even with this relatively weak constraint, we found a robust shortlist effect. This makes us hopeful that a similar effect could be found in collections where size is more directly constrained by strong material limitations, like physical collections of books, artwork, etc.

The shortlist effect allows us to observe the selective value of a cultural item in a way that decouples it from the item's frequency. With drift or conformity-biased transmission, the success of cultural items is influenced by their frequency in a population (i.e. how widely they are adopted), either through unbiased copying (agents are more likely to encounter more prevalent items, and thus, to acquire them), or through conformity-biased copying (agents are more likely to acquire more prevalent items once they encounter them, because they wish to imitate the majority). This has led to the suggestion that intrinsic appeal matters little to cultural success in real-world datasets, in spite of experimental (alongside anecdotal) evidence for its existence. Adding content-biased selection to these hypotheses allows us to consider the possibility that some ‘hidden gem’ items have not reached high frequency (owing to drift or conformity), but are still quite likely to be adopted if encountered. A typical hidden gem in our MovieLens dataset is the cult classic *Primer* by Shane Carruth (2004) – a relatively poorly known movie revered by those who understand it. *Primer* is highly rated but present in few MovieLens collections. Its contribution to the nestedness of the MovieLens sci-fi corpus is among the lowest. Conversely, some items may be ‘accidental hits’ that owe their prevalence to drift and conformity, without actually being appealing to most viewers.

Yet both hidden gems and accidental hits remain difficult to detect in real-world data, because the effects of intrinsic appeal on cultural diffusion are difficult to disentangle from those of mere drift or selection based on conformity. Even when a plausible link can be established between the usefulness of a technology, or the appeal of an item, and their prevalence (Acerbi & Bentley, 2014; Steele et al., 2010), quantitative models cannot prove this conclusively. Understandably, then, the main effort has been to distinguish drift from selection based on conformity or its variants (Acerbi & Bentley, 2014; Bentley, 2008; Crema et al., 2016; Kandler & Shennan, 2013; Mesoudi & Lycett, 2009). This is usually done by considering population-level statistics, for instance the frequency distribution of variants, and inferring the importance of selectionist vs. neutral dynamics therefrom (Carrignon et al., 2019; Crema et al., 2014; Kandler & Powell, 2018). These cutting-edge methods are crucial in detecting the impact of selectionist dynamics over entire populations of cultural items.

Our study goes beyond the state of the art by showing how we can detect the contribution of appeal to the success of individual movies, by looking at the contribution that each movie makes to the nestedness of a set of collections. Having first argued, using a model, that stronger cultural selection results in higher nestedness at the population level, we proceeded to show, empirically, that individual movies contributed to the nestedness of movie collections to the extent that their frequency matched their appeal. We also empirically verified the main assumption that guided our work: the shortlist effect. Small collections are more selective for high-appeal items. Both results were obtained by testing pre-registered predictions on 28 different datasets.

The shortlist effect could be exploited to spot ‘accidental hits’ and ‘hidden gems’ in large cultural collections, even in ancient data where any information on human tastes and preferences has been lost. The method used here is quite general and requires only thin data, of a format familiar to ecologists, anthropologists and archaeologists. It could allow us to determine the appeal of cultural items in a broad range of domains, from popular culture to scientific papers or archaeological vestiges. The shortlist effect can be estimated from any dataset that consists of a series of collections, that is to say, a series of datasets characterised by the presence or absence of certain cultural items, similar to the presence/absence matrices used in ecology. Technological repertoires (as studied by Kamilar & Atkinson, 2014), graphical repertoires in pottery (Kandler & Shennan, 2013; Shennan & Wilkinson, 2001) or carpet weaving (Tehrani & Collard, 2002) could thus be studied quantitatively to reveal past cultural preferences.

## Materials and methods

### Simulations of nestedness in sets of collections, with and without content-biased selection and the shortlist effect

We simulated presence/absence matrices (200 by 200 cells) representing the presence or absence of items (columns) in collections (rows). A cell takes a value of 1 if the item is present in the collection, 0 otherwise. Values are determined using equation (1). Three parameters are made to vary: the strength of the correlation between item frequency and appeal; the importance of the shortlist effect, i.e. the value of the s parameter; and the basic likelihood that items will occur, regardless of their appeal (the *e* factor in the equation). Each collection's carrying capacity (*k*) was drawn from a continuous distribution between 0 and 1. This was also the case for item frequencies and item appeals. Item appeals were fixated so they would correlate with item frequencies at the desired levels, using the *fabricatr* package for R (Blair et al., 2019). We tested the following parameter values:

- *e*: {0.0000001, 0.001, 0.01, 0.1, 0.25, 0.5, 0.75, 1};
- *s*: {0.0000001, 0.001, 0.01, 0.1, 0.25, 0.5, 0.75, 1};
- the frequency–appeal correlation: {0, 0.25, 0.50, 0.75, 1}.

Crossing these parameter values, we get 320 conditions. Thirty simulations were run for each condition. We used NODF to measure nestedness (Almeida-Neto et al., 2008; Ulrich et al., 2009), the most common measure (computed with the R package *vegan*; Oksanen et al., 2019). We considered the average of the NODF values over the 30 simulations (Table 1). We get a perfect or near-perfect correlation (*r* > 0.95) whenever *s* and *e* are of the same order of magnitude. The correlation is substantial (*r* > 0.70) whenever *s* > *e*. The relationship breaks down (*r* < 0.5) only when *s* is much smaller than *e* (25 times smaller at least). None of these results change if we control for matrix fill, i.e. the degree to which the matrix is full as opposed to empty (NODF is known to be confounded with matrix fill to an important extent; Ulrich et al., 2009).

### Preregistration

All of the analyses concerning the Netflix dataset were preregistered on the Open Science Framework (predictions, measurements, statistical models, sample size and exclusion criteria). The results for the MovieLens datasets were arrived at in an exploratory fashion, in the course of testing a slightly different hypothesis, hence we decided to replicate them with the Netflix datasets. This applies to all results described here. The one exception is the analysis of the shortlist effect in the Netflix dataset, where our pre-registered threshold of 50,000 users had to be lowered for computational reasons (see below). Several registrations were made, allowing us to test follow-up hypotheses. Our complete research diary, available at https://osf.io/qem78/, is an exhaustive record of all of the registration documents and all of the analyses that were run.

### Data selection

We selected subsets from two large-scale movie collections, the MovieLens (c. 138,000 unique users, 27,000 different films) and Netflix Prize datasets (480,189 unique users, 18,106 different films). We divided each dataset into subsets based on genre, then sampled from these subsets. The sampling differed depending on the dataset and analysis. Measuring nestedness contributions is computationally intensive and can only be done on relatively small datasets, whereas our analyses bearing on the shortlist effect, being less heavy, can accommodate more data. Supplementary Tables S1 and S2 give the detail of data selection for each dataset and analysis. All data exclusions were made in advance of data analysis and, with one exception (described below), were run as preregistered.

Genre subsets were determined, for both datasets, using genre tags (‘drama’, ‘comedy’, ‘horror’, etc.) present in the data (for MovieLens) or from the IMDb database (for Netflix). This produced 13 (Netflix) to 15 (MovieLens) non-overlapping subsets. Most films carry more than one genre tag (e.g. horror + thriller, horror + mystery + thriller), and some tags are much more prevalent than others. Our goal was to make the genre subsets as similar in size as possible, while avoiding any overlap between them. Thus, our inclusion criteria were stringent for frequent genres and less so for under-represented genres. After this step, we excluded from the Netflix dataset all of the movies that did not have a rating in both the IMDb and the MovieLens sources.

To run the analyses bearing on the shortlist effect, we had to limit the subsets’ sizes, for computational reasons. For the MovieLens dataset, we limited the number of collections to 50,000 (maximum), randomly drawn from the existing collections. Since most Netflix subsets contain much greater numbers of movies than the MovieLens subsets, this threshold had to be lowered to 20,000 for the Netflix analysis to be tractable (this is the only time we had to depart from our preregistered criteria for data exclusions).

To run the nestedness contributions analysis on the MovieLens dataset, we took the genre subsets and kept only the 10% of users with the largest film collections and the 70% most widespread films. For the Netflix subsets, we discarded the infrequent films (*n* ≤ 15 user ratings) and sampled the ratings of 500 randomly selected users.

### NODF measure for nestedness

The nestedness measure based on Overlap and Decreasing Fill is one of the standard measures for nestedness (Almeida-Neto et al., 2008; Ulrich et al., 2009). It requires an ordered matrix of interactions: in our case, a matrix where rows represent collections and columns stand for movies. A cell takes the value 1 if the (column) movie is present in the (row) collection and 0 otherwise. The matrix is ordered so that the most populated rows (i.e. more 1 values) are on top, and the most populated columns are on the left. The NODF algorithm compares every adjacent pair of columns and every adjacent pair of rows, from top to bottom and from right to left. The comparison follows two criteria. The first is *decreasing fill*: the row on the bottom (for columns, the column on the right) must be less populated than the row on top (for columns, the column on the left). Any pair of rows or columns that fails to show decreasing fill gets a pair score of 0. For pairs of rows or columns that do show decreasing fill, NODF applies the second criterion: *overlap*. It counts how many 1 values in the bottom row (right column) overlap with a 1 value in the top row (left column). The score assigned to a pair of rows or columns is the percentage of full cells (1 value) that are so aligned. Pair scores are averaged over all pairs of rows, then over all pairs of columns. The average of these two averages is the final NODF score.

### Nestedness contributions

We used Saavedra et al.'s (2011) measure of nestedness contribution, given by equation (2):2
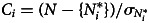
where *C_i_* is the contribution of an item *i* to the nestedness of a set of collections, *N* is the actual nestedness value, {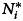} is the average value of *N* for a set of simulations where the presence of item *i* in the set's collections has been randomised, preserving the true values for all other items (see below), and 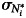
is the standard deviation around this average. Randomisations of the item's presence in collections were generated according to equation (3):3
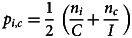

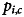
 being the probability that the randomisation will determine item *i* to be present in collection *c*, *n_i_* being the total number of collections that item *i* is present in, *n_c_* the total number of items in collection *c*, and *I* and *C* being the total number of items and collections (respectively) in the set.

### Significance estimate for NODF measures

Computing the significance of our measures of nestedness for each of the 28 movie corpora involved generating randomly populated matrices, and comparing their NODF scores with those of the real matrix. The value of such an analysis hinges on the particular randomisation procedure, or null hypothesis, that one adopts. Following the classification of Mariani et al. (2019), we decided to avoid the least constrained models and the most constrained model, focusing on a middle ground option: the column proportional–row-fixed model (‘R1 method’ in the vegan package for R). This model generates a matrix identical in size to the original matrix, with each row also having the same number of full cells (i.e. cells with a value of 1) as in the original matrix, but with the location of full cells inside each row being randomised. The randomisation is based on the probability, for each column, of having full cells (in the original matrix). Our significance tests are based on 200 such simulations for each corpus.

## Supplementary material

For supplementary material accompanying this paper visit https://doi.org/10.1017/ehs.2021.48. click here to view supplementary material

## Research transparency and reproducibility

We are not allowed to disseminate the Netflix and MovieLens datasets publicly. We provide the complete R code that we used on the MovieLens dataset, which is publicly available. Please get in touch with the corresponding author for more detail. The replication on the Netflix dataset used an identical procedure. We provide a detailed table of our results for both datasets. All of our material is available at https://osf.io/qem78/
