# Supplementary material for: The shortlist effect: nestedness contributions as a tool to explain cultural success
Source: Evol Hum Sci. 2021 Nov 8;3:e51. doi: 10.1017/ehs.2021.48 (PMC10427280; doi:10.1017/ehs.2021.48)
Supplement: Supplementary file 1 [file S2513843X21000487sup.zip › S2513843X21000487sup001.docx]

**The Shortlist Effect: Nestedness contributions as a tool to explain cultural success**

**Supplementary material**

|  | Users / movies after Step 1 (genre-based subsets constitution) | Users / movies after Step 2 (discarding the 90% smallest collections and 30% least frequent movies) | Dataset used for the Shortlist Effect (picking up to 50,000 random collections) |
| --- | --- | --- | --- |
| action | 91,227 | 600 | 50,000 |
|  | 784 | 117 (91) | 117 |
| adventure | 109,876 | 1329 | 50,000 |
|  | 322 | 42 (39) | 42 |
| animation | 59,503 | 550 | 50,000 |
|  | 391 | 51 (36) | 51 |
| comedy | 123,031 | 736 | 50,000 |
|  | 2,251 | 306 (246) | 306 |
| crime | 120,404 | 951 | 50,000 |
|  | 1,111 | 158 (142) | 158 |
| documentary | 45,190 | 174 | 44516 |
|  | 1,879 | 290 (192) | 290 |
| drama | 122,294 | 565 | 50,000 |
|  | 4,416 | 633 (439) | 633 |
| fantasy | 96,666 | 1115 | 50,000 |
|  | 402 | 59 (51) | 59 |
| horror | 57,801 | 250 | 50,000 |
|  | 1,042 | 192 (156) | 192 |
| noir | 25,407 | 167 | 25077 |
|  | 216 | 49 (18) | 49 |
| romantic comedy | 113,903 | 929 | 50,000 |
|  | 741 | 128 (107) | 128 |
| science fiction | 97,554 | 763 | 50,000 |
|  | 592 | 87 (70) | 87 |
| thriller | 113,575 | 795 | 50,000 |
|  | 866 | 137 (115) | 137 |
| war | 105,098 | 1039 | 50,000 |
|  | 626 | 75 (62) | 75 |
| western | 60,598 | 479 | 50,000 |
|  | 449 | 61 (36) | 61 |

**Table S1. Constitution of the genre subsets (MovieLens).** Number of users included in the genre subsets of the MovieLens dataset, for each step of the analysis. The original dataset includes around 20 million ratings of 27,000 films by 138,000 unique users. We broke it down into 15 genre-based subsets (first and second column). The resulting number of users is given in the top cell, the number of movies in the bottom cell. The third column gives the number of movies and users for the analysis bearing on drift scores and nestedness contributions (see Fig. 4). The figure between parentheses indicates the number of movies analyzed with ratings from the Metacritic source. The last column indicates the number of users and movies that were analyzed for our study of the shortlist effect (see Figure 3).

|  | Users / movies after Step 1 (genre-based corpora constitution) | Users / movies after Step 2  (a random sample of 500 users) | Dataset used for the Shortlist Effect (up to 20,000 random collections) |
| --- | --- | --- | --- |
| action | 383,940 | 500 | 20,000 |
|  | 463 | 375 | 375 |
| adventure | 348,911 | 500 | 20,000 |
|  | 372 | 333 | 333 |
| biographical | 389,068 | 500 | 20,000 |
|  | 290 | 277 | 277 |
| comedy | 387,037 | 500 | 20,000 |
|  | 501 | 434 | 434 |
| drama | 350,234 | 500 | 20,000 |
|  | 422 | 379 | 379 |
| family | 374,178 | 500 | 20,000 |
|  | 344 | 327 | 327 |
| fantasy | 383,107 | 500 | 20,000 |
|  | 281 | 253 | 253 |
| historical | 303,875 | 500 | 20,000 |
|  | 241 | 229 | 229 |
| horror | 252,513 | 500 | 20,000 |
|  | 415 | 391 | 391 |
| musical | 328,175 | 500 | 20,000 |
|  | 364 | 340 | 340 |
| romance | 341,640 | 500 | 20,000 |
|  | 364 | 341 | 341 |
| science fiction | 375,509 | 500 | 20,000 |
|  | 370 | 321 | 321 |
| thriller | 439,032 | 500 | 20,000 |
|  | 598 | 493 | 493 |

**Table S2. Constitution of the genre subsets (Netflix).** Number of users included in the genre subsets of the Netflix dataset, for each step of the analysis. The original dataset includes around 100 million ratings from 480,189 unique users, rating 17,770 unique items (movies). We broke it down into 13 genre-based subsets (first and second column). The resulting number of users is given in the top cell, the number of movies in the bottom cell. The third column gives the number of movies and users for the analysis bearing on drift scores and nestedness contributions (see Fig. 4). The last column indicates the number of users and movies that were analyzed for our study of the shortlist effect (see Figure 3).

| **Genre subset** | **Ratings source** | **Frequency-ratings correlation (Spearman’s rho)** | **p-value** | **95% C.I. (lower bound)** |
| --- | --- | --- | --- | --- |
| **action** | **MovieLens** | 0.19 | 0.003 | 0.046 |
|  | **IMDb** | 0.11 | 0.22 | -0.050 |
|  | **Metacritic** | 0.18 | 0.0008 | 0.018 |
| **adventure** | **MovieLens** | 0.20 | 0.18 | -0.053 |
|  | **IMDb** | 0.30 | 0.04 | 0.036 |
|  | **Metacritic** | 0.37 | 0.01 | 0.162 |
| **animation** | **MovieLens** | 0.40 | 0.003 | 0.198 |
|  | **IMDb** | 0.35 | 0.01 | 0.131 |
|  | **Metacritic** | 0.36 | 0.02 | 0.059 |
| **comedy** | **MovieLens** | 0.26 | < .00001 | 0.172 |
|  | **IMDb** | 0.32 | < .00001 | 0.238 |
|  | **Metacritic** | 0.29 | < .00001 | 0.190 |
| **crime** | **MovieLens** | 0.37 | < .00001 | 0.253 |
|  | **IMDb** | 0.45 | < .00001 | 0.330 |
|  | **Metacritic** | 0.17 | 0.03 | 0.041 |
| **documentary** | **MovieLens** | 0.56 | < .00001 | 0.496 |
|  | **IMDb** | 0.24 | < .00001 | 0.153 |
|  | **Metacritic** | 0.24 | < .00001 | 0.135 |
| **drama** | **MovieLens** | 0.24 | < .00001 | 0.178 |
|  | **IMDb** | 0.21 | < .00001 | 0.152 |
|  | **Metacritic** | 0.18 | < .00001 | 0.106 |
| **fantasy** | **MovieLens** | 0.49 | < .00001 | 0.292 |
|  | **IMDb** | 0.55 | < .00001 | 0.389 |
|  | **Metacritic** | 0.49 | 0.0002 | 0.286 |
| **horror** | **MovieLens** | 0.06 | 0.36 | -0.059 |
|  | **IMDb** | 0.15 | 0.02 | 0.026 |
|  | **Metacritic** | 0.13 | 0.08 | -0.006 |
| **noir** | **MovieLens** | 0.51 | 0.0001 | 0.293 |
|  | **IMDb** | 0.20 | 0.14 | -0.041 |
|  | **Metacritic** | 0.30 | 0.21 | -0.183 |
| **romantic comedy** | **MovieLens** | 0.41 | < .00001 | 0.295 |
|  | **IMDb** | 0.41 | < .00001 | 0.288 |
|  | **Metacritic** | 0.38 | < .00001 | 0.242 |
| **science fiction** | **MovieLens** | 0.27 | 0.01 | 0.104 |
|  | **IMDb** | 0.25 | 0.01 | 0.072 |
|  | **Metacritic** | 0.28 | 0.01 | 0.085 |
| **thriller** | **MovieLens** | 0.27 | 0.001 | 0.134 |
|  | **IMDb** | 0.29 | 0.0003 | 0.153 |
|  | **Metacritic** | 0.10 | 0.27 | -0.062 |
| **war** | **MovieLens** | 0.31 | 0.006 | 0.133 |
|  | **IMDb** | 0.46 | < .00001 | 0.284 |
|  | **Metacritic** | 0.41 | 0.0008 | 0.198 |
| **western** | **MovieLens** | 0.44 | 0.0003 | 0.257 |
|  | **IMDb** | 0.36 | 0.003 | 0.153 |
|  | **Metacritic** | 0.11 | 0.50 | -0.195 |

**Table S3. Content-biased selection in the Movielens dataset.** Reported values are truncated, not rounded.

| **Genre subset** | **Ratings source** | **Frequency-ratings correlation (Spearman’s rho)** | **p-value** | **95% C.I. (lower bound)** |
| --- | --- | --- | --- | --- |
| **action** | **MovieLens** | 0.12 | .001 | 0.04 |
|  | **IMDb** | 0.16 | .0001 | 0.07 |
|  | **Netflix** | 0.40 | < .00001 | 0.37 |
| **adventure** | **MovieLens** | 0.13 | .001 | 0.04 |
|  | **IMDb** | 0.18 | < .0001 | 0.09 |
|  | **Netflix** | 0.32 | < .00001 | 0.24 |
| **biography** | **MovieLens** | 0.37 | < .00001 | 0.28 |
|  | **IMDb** | 0.36 | < .00001 | 0.27 |
|  | **Netflix** | 0.55 | < .00001 | 0.48 |
| **comedy** | **MovieLens** | 0.20 | < .00001 | 0.13 |
|  | **IMDb** | 0.22 | < .00001 | 0.15 |
|  | **Netflix** | 0.49 | < .00001 | 0.43 |
| **drama** | **MovieLens** | 0.39 | < .00001 | 0.31 |
|  | **IMDb** | 0.39 | < .00001 | 0.31 |
|  | **Netflix** | 0.59 | < .00001 | 0.53 |
| **family** | **MovieLens** | 0.11 | .003 | 0.02 |
|  | **IMDb** | 0.12 | .002 | 0.03 |
|  | **Netflix** | 0.34 | < .00001 | 0.25 |
| **fantasy** | **MovieLens** | 0.28 | < .00001 | 0.18 |
|  | **IMDb** | 0.25 | < .00001 | 0.14 |
|  | **Netflix** | 0.55 | < .00001 | 0.46 |
| **historical** | **MovieLens** | 0.34 | < .00001 | 0.24 |
|  | **IMDb** | 0.47 | < .00001 | 0.39 |
|  | **Netflix** | 0.63 | < .00001 | 0.56 |
| **horror** | **MovieLens** | 0.29 | < .00001 | 0.21 |
|  | **IMDb** | 0.20 | < .00001 | 0.12 |
|  | **Netflix** | 0.60 | < .00001 | 0.55 |
| **musical** | **MovieLens** | 0.06 | .02 | - 0.03 |
|  | **IMDb** | 0.14 | .006 | 0.05 |
|  | **Netflix** | 0.33 | < .00001 | 0.25 |
| **romance** | **MovieLens** | 0.17 | .0001 | 0.08 |
|  | **IMDb** | 0.26 | < .00001 | 0.18 |
|  | **Netflix** | 0.52 | < .00001 | 0.45 |
| **science fiction** | **MovieLens** | 0.50 | < .00001 | 0.43 |
|  | **IMDb** | 0.41 | < .00001 | 0.33 |
|  | **Netflix** | 0.60 | < .00001 | 0.53 |
| **thriller** | **MovieLens** | 0.37 | < .00001 | 0.30 |
|  | **IMDb** | 0.31 | < .00001 | 0.25 |
|  | **Netflix** | 0.60 | < .00001 | 0.55 |

**Table S4. Content-biased selection in the Netflix dataset.** Reported values are truncated, not rounded.

| **corpus** | ICC  for the 3 sets of ratings | ICC  without Metacritic ratings |
| --- | --- | --- |
| action | 0.587 | 0.904 |
| adventure | 0.703 | 0.895 |
| animation | 0.712 | 0.773 |
| comedy | 0.697 | 0.871 |
| crime | 0.766 | 0.895 |
| documentary | 0.633 | 0.756 |
| drama | 0.635 | 0.804 |
| fantasy | 0.856 | 0.961 |
| horror | 0.686 | 0.829 |
| noir | 0.686 | 0.832 |
| romcom | 0.749 | 0.913 |
| scifi | 0.755 | 0.904 |
| thriller | 0.745 | 0.928 |
| war | 0.645 | 0.65 |
| western | 0.559 | 0.86 |

**Table S5. Consistency between sets of ratings of the MovieLens corpus.** For each corpus, we computed the intraclass correlation (ICC) for the ratings given by our three sets of ratings (IMDb, MovieLens, and Metacritic) for each of the movies in the corpus. A second ICC (third column) was computed using only the MovieLens and Metacritic ratings, because we suspected that Metacritic ratings (produced exclusively by professional film critics, unlike the other ratings) were markedly different from the rest of the ratings. Both ICCs examine consistency, not agreement, for single scores, not averages. The first ICC uses a one-way model (because some Metacritic scores are NA), the second uses a two-way model (because all movies have both a Movielens score and an IMDb one). All ICCs were computed with the irr package for R, using min-max normalized ratings.

| **corpus** | ICC  for the 3 sets of ratings |
| --- | --- |
| action | 0.654 |
| adventure | 0.785 |
| biopic | 0.685 |
| comedy | 0.711 |
| drama | 0.712 |
| family | 0.733 |
| fantasy | 0.761 |
| historical | 0.591 |
| horror | 0.737 |
| musical | 0.705 |
| romance | 0.611 |
| scifi | 0.792 |
| thriller | 0.699 |

**Table S6. Consistency between sets of ratings of the Netflix corpus.** For each corpus, we computed the intraclass correlation for the ratings given by our three sets of ratings (IMDb, MovieLens, and Netflix) for each of the movies in the corpus. The ICC examine consistency, not agreement, for single scores, not averages, using a two-way model (because all movies have a score for all sets of rating). All ICCs were computed with the irr package for R, using min-max normalized ratings.

**Fig. S1.** Histograms of the distribution of ratings for the two corpora, MovieLens (a) and Netflix (b). The y axis shows the number of individual ratings given by participants for a given value. The irregularities in the MovieLens distribution are due to the fact that users tend to avoid giving half-star ratings (1.5, 2.5, etc.).
