# Supplementary material for: The shortlist effect: nestedness contributions as a tool to explain cultural success
Source: Evol Hum Sci. 2021 Nov 8;3:e51. doi: 10.1017/ehs.2021.48 (PMC10427280; doi:10.1017/ehs.2021.48)
Supplement: Supplementary file 1 [file S2513843X21000487sup.zip › S2513843X21000487sup002.docx]

|  | **Values of s** | | | | | | | | |
| --- | --- | --- | --- | --- | --- | --- | --- | --- | --- |
| **Values of e** |  | **0.0000001** | **0.001** | **0.01** | **0.1** | **0.25** | **0.5** | **0.75** | **1** |
|  | **0.0000001** | **0.99 / 0.99** | **0.97 / 0.90** | 0.94 / 0.87 | **0.88 / 0.90** | 0.88 / 0.73 | **0.93 / 0.94** | **0.92 / 0.90** | 0.85 / 0.84 |
|  | **0.001** | 0.91 / 0.69 | **0.99 / 0.99** | **0.99 / 0.99** | **0.99 / 0.99** | **0.97 / 0.97** | 0.82 / 0.85 | 0.75 / 0.86 | 0.91 / 0.71 |
|  | **0.01** | 0.75 / 0.70 | 0.92 / 0.77 | **0.99 / 0.99** | **0.99 / 0.99** | **0.99 / 0. 98** | **0.99 / 0.98** | **0.98 / 0.94** | 0.96 / 0.83 |
|  | **0.1** | 0.76 / 0.79 | 0.72 / 0.26 | **0.99 / 0. 94** | **0.99 / 0.99** | **0.99 / 0.99** | **0.99 / 0.99** | **0.99 / 0.99** | **0.99 / 0.99** |
|  | **0.25** | -0.15 / 0.26 | 0.40 / 0.34 | 0.01 / -0.42 | **0.99 / 0.99** | **0.99 / 0.99** | **0.99 / 0.99** | **0.99 / 0.99** | **0.99 / 0.99** |
|  | **0.5** | 0.87 / 0.68 | 0.93 / 0.92 | -0.71 / 0.32 | **0.99 / 0.99** | **0.99 / 0.99** | **0.99 / 0.99** | **0.99 / 0.99** | **0.99 / 0.99** |
|  | **0.75** | 0.07 / -0.32 | 0.54 / 0.55 | 0.90 / 0.24 | 0.98 / 0.89 | **0.99 / 0.99** | **0.98 / 0.99** | **0.99 / 0.99** | **0.99 / 0.99** |
|  | **1** | 0.59 / 0.58 | -0.04 / 0.38 | -0.42 / 0.54 | **0.98 / 0.96** | **0.99 / 0.98** | **0.99 / 0.97** | **0.98 / 0.99** | **0.99 / 0.99** |
